# Virtual reality-based rehabilitation experience of stroke survivors: a meta-synthesis of qualitative studies

**DOI:** 10.3389/fpubh.2025.1566813

**Published:** 2025-07-24

**Authors:** Jingyi Cai, Beixue Liu, Mingyang Song, Qiuyi Wang, Lanshu Zhou

**Affiliations:** ^1^Key Laboratory of Geriatric Long-term Care (Naval Medical University), Ministry of Education, Shanghai, China; ^2^Department of Nursing, Longhua Hospital Affiliated to Shanghai University of Traditional Chinese Medicine, Shanghai, China; ^3^Department of Nursing Clinical Nursing Teaching and Research, Naval Medical University, Shanghai, China

**Keywords:** stroke, virtual reality, rehabilitation, experience, meta-synthesis

## Abstract

**Aim:**

To examine and synthesize qualitative data regarding the experiences of stroke survivors who underwent rehabilitation using virtual reality (VR).

**Methods:**

We critically appraised the included study using the Joanna Briggs Institute Critical Assessment Checklist for Qualitative Research. Relevant qualitative studies were searched in PubMed, EMBASE, CINAHL, Web of Science, Cochrane Library, China National Knowledge Infrastructure, Wan Fang Database, China Science and Technology Journal Database, and China Biomedical Literature Database from inception to December 2024. Two researchers independently extracted and analysed the data and integrated the results using a pooled meta-aggregation approach.

**Results:**

Sixteen studies (*n* = 145 stroke survivors) were included. A total of 5 meta-themes, 12 categories, and 38 research results were extracted. The meta-themes included perceived benefits, facilitating factors of using VR, and hindering factors of using VR, changes in the physical and mental health following VR rehabilitation, and suggestions and expectations for VR rehabilitation.

**Conclusion:**

Stroke survivors perceived the benefits and barriers of using VR-based rehabilitation (VRBR); therefore, it is a complex perceptual change for them. They require emotional support from family and peers, professional support from healthcare professionals, and financial support from the government. Moreover, rehabilitation motivation is an important factor that influences utilization. Updating VRBR content and functionality is essential to improve user experience.

**Systematic review registration:**

PROSPERO CRD42024504700, https://www.crd.york.ac.uk/PROSPERO/search.

## 1 Introduction

Stroke, a leading cause of death and disability (1), incurs a global cost estimated at over US$721 billion, which is 0.66% of the global GDP (2). Rehabilitation therapy effectively reduces disability in stroke survivors (3). In China alone, approximately 25 million stroke survivors required rehabilitation therapy in 2019 (4). Traditional rehabilitation treatments include physical therapy (5), speech therapy (6) and occupational therapy (7). New assistive technologies for rehabilitation therapy, such as virtual reality (VR), rehabilitation robots, and remote rehabilitation technology (8), are valued for their convenience and task-oriented training. VR combines computer graphics technology and related equipment, providing immersive and interactive virtual environments for patients and obtaining real-time feedback on their performance through multiple senses (9). Virtual reality-based rehabilitation (VRBR) can be tailored to a patient’s needs and is a personalized and progressive rehabilitation method (10). Currently, numerous quantitative studies have confirmed the effectiveness of VRBR on the upper limb (11), lower limb (12), and cognitive functions (13) of stroke survivors. However, qualitative research is needed to explore the motivation for rehabilitation, as well as facilitators and barriers to using VRBR. Qualitative meta-synthesis combines qualitative findings from disparate investigations (14) and meta-synthesis of existing qualitative studies, improving the generalization and resonance of the results. This study aimed to systematically evaluate the experiences and needs of individuals who survived a stroke when using VRBR to exercise upper limb, lower limb, balance, walking, and cognitive functions. This meta-synthesis of qualitative studies will propose specific strategies to improve VRBR interventions, providing a reference for future research on VRBR for stroke survivors.

## 2 Literature review

Previous studies have shown that although stroke patients have no preference for user interfaces (15), interesting and immersive virtual reality scenarios not only appeal to young stroke patients, but also have the potential to maintain stroke patients’ motivation for rehabilitation (16). In addition, virtual reality-based rehabilitation therapy has been experienced better in patients with other diseases. For instance, breast cancer patients had high levels of acceptance and satisfaction with VRBR interventions and are willing to recommend this approach to their peers (17). Finally, three key concepts mentioned in this study are explained here to the reader. VRBR is an innovative therapeutic approach utilizing virtual reality technology, which integrates motor learning, neuroplasticity, and ecological systems theory to deliver a dynamic, patient-centered, and task-oriented rehabilitation program (e.g., for stroke survivors) (10). Virtual reality-based rehabilitation (VRBR) employs immersive, task-specific virtual environments to promote motor learning and neuroplasticity through adaptive training paradigms, real-time biofeedback, and ecologically valid simulations that enhance skill transfer to daily living while maintaining patient engagement and personalized therapeutic challenge (18). Rehabilitation motivation refers to the psychological need for individuals to engage in exercise and therapy for rehabilitation (19). Social support in rehabilitation is defined as a comprehensive system of external factors, including family members, friends, healthcare professionals, and government financial support, that help individuals cope with illness or injury and promote their psychological and physical well-being (20).

## 3 Methods

### 3.1 Reporting guidelines

This study was conformed to the ENTREQ (Enhancing Transparency in Reporting the Synthesis of Qualitative Research) statement, which can improve the transparency of qualitative research synthesis reports (21). The search results were tracked and reported according to the Preferred Reporting Items for Systematic Reviews and Meta-Analysis (PRISMA) standards (22).

### 3.2 Inclusion and exclusion criteria

The inclusion criteria for the literature were determined according to the PICoS principles (23). Inclusion criteria: (a) study population (P): individuals who survived a stroke (age ≥18 years; non-acute phase); (b) phenomenon of interest (I): attitudes, experiences, and expectations of VRBR; (c) context (Co): experiences with VRBR in the hospital, community, or home; (d) study design (S): qualitative research, including phenomenological studies, descriptive qualitative research, rooted theory, ethnography.

Exclusion criteria: (a) duplicate publications (retaining only the one with the most information); (b) literature for which full text was not available, or data were incomplete; (c) literature not in Chinese or English; and (d) literature designed using mixed methods and qualitative data that cannot be separated.

### 3.3 Data sources and search strategy

PubMed, Embase, CINAHL, Web of Science, Cochrane Library, China National Knowledge Infrastructure, Wan Fang Database, China Science and Technology Journal Database, and China Biomedical Literature Database were searched using a computer, and the retrieval time limit was from inception to December 2024. The words are retrieved in the following form: stroke (stroke*, cerebrovascular*, accident*, cerebral infarction*, cerebral haemorrhage*, apoplexy*, or poststroke*), virtual reality rehabilitation (virtual reality*, exergames*, gaming*, serious games*, motion-sensing games*, or interactive games*, virtual therapy*,or virtual rehabilitation*), qualitative (qualitative*, interview*, attitude*, experience*, phenomenology*, qualitative research*, qualitative thematic analysis*, semi-structured*). Additionally, we traced the reference lists of the included studies to identify potentially eligible studies.

### 3.4 Literature selection

The literature was imported into EndnoteX9.1, and duplicates were removed. The literature was independently screened by two researchers trained in evidence-based nursing, who cross-checked the extracted information. In cases of disagreement, a third researcher was consulted to assist in the judgment process. The extracts included authors, year, country, methodology, data collection, data analysis, study population, phenomena of interest, and main results.

### 3.5 Literature quality assessment

According to the Australian Joanna Briggs Institute Center for Evidence-Based Health Care’s (2016) quality assessment criteria for qualitative research, two researchers independently evaluated the methodology of the included literature. The assessment consisted of 10 elements, each rated as “yes,” “no,” “unclear,” or “not applicable.” The quality of the included literature was categorized into three grades: A (met all evaluation indicators), B (partially met), and C (did not meet). Studies with grade C were excluded. When disagreements occurred, the two researchers made decisions based on mutual agreement. If necessary, a third researcher included the literature.

### 3.6 Data analysis

In this study, the results were integrated using the meta-aggregation approach (23), which requires a three-step approach: (1) extracting all findings from the included studies with an accompanying illustration and an allocated level of plausibility for each finding; (2) grouping findings into categories, with at least two findings per category; and (3) synthesizing one or more findings from at least two categories. The results were thoroughly understood and meticulously analysed multiple times by two researchers to ensure accuracy and depth of interpretation. They grouped similar results into new categories, which were summarized into meta-themes.

### 3.7 Meta-theme results quality evaluation

The meta-theme results were rated for quality (high, medium, low, and very low) according to the Qualitative Research Integration Evidence Assessment Tool (confidence in the qualitative evidence, ConQual) (24) constructed by the JBI Center for Evidence-Based Health Care. Ratings began at “high” and were downgraded based on reliability and trustworthiness. Dependability was determined using five questions from the JBI Quality Evaluation Criteria for Qualitative Research, which mainly evaluated the quality of the original studies included in the meta-analysis. A result of 4–5 positive answers indicated “not downgraded,” 2–3 positive answers indicated “downgraded 1,” and 0–1 positive answer indicated “downgraded 2.” Credibility was examined for consistency between the integrated results (i.e., the authors’ interpretations) and the supporting data. The findings were rated on three levels: unequivocal, equivocal, and unsupported. “No downgrade” if the integrated evidence is all from unequivocal results; ‘downgrade 2’ if the integrated evidence is all from equivocal results; and ‘downgrade 4’ if the integrated evidence is all from unsupported results”.

## 4 Results

### 4.1 Results of the literature search

**Figure 1:**
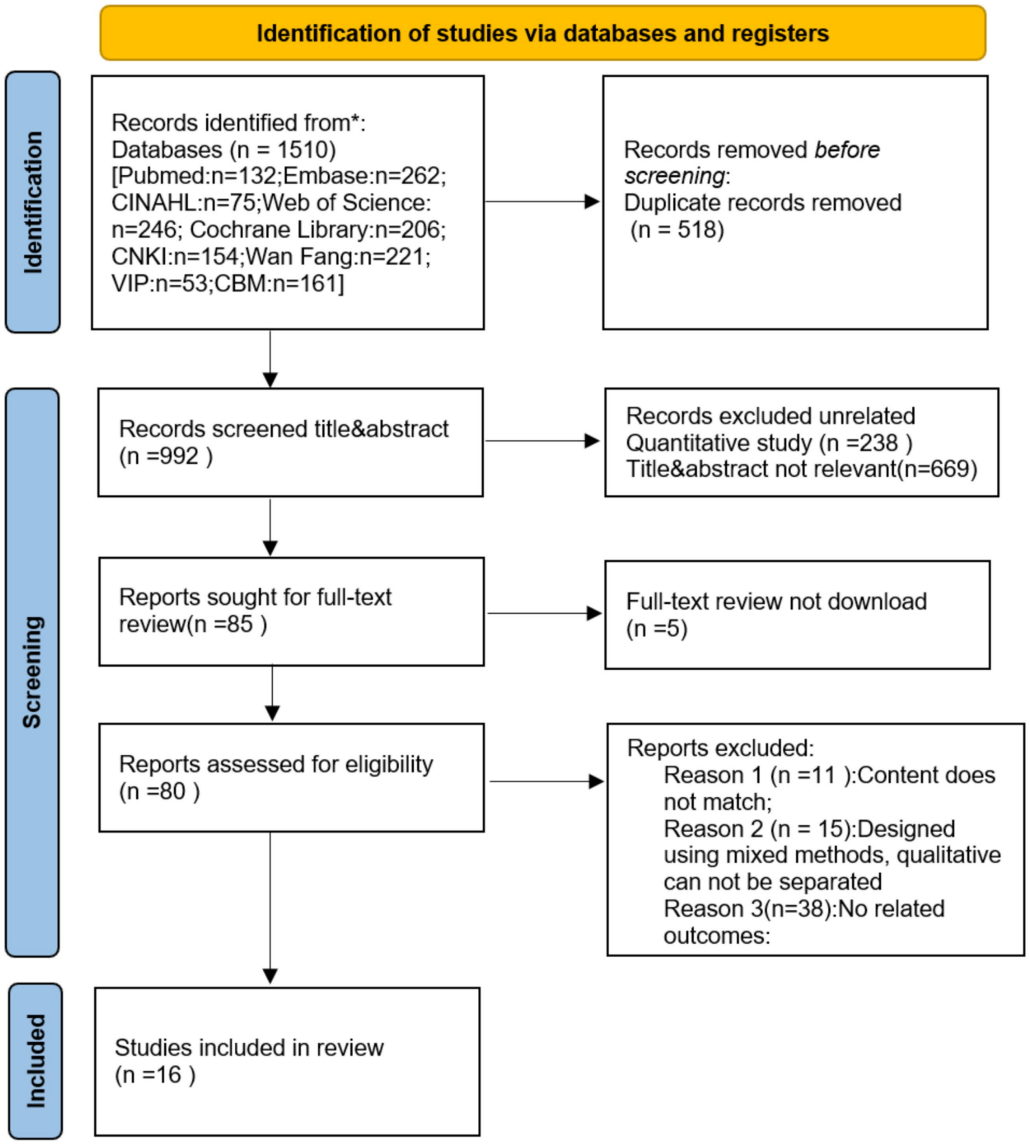
Flow chart of literature screening.

Our researchers initially examined 1,510 articles in the literature, ultimately retaining 16 literature. A detailed flowchart of the literature review is shown in Figure 1. Table 1 summarizes the features of the included studies.

**Table 1:** Characteristics of included literatures.

Authors, year, country	Methodology	Data collection	Data analysis	Study population	Phenomenon of interest	Main results
Suai Li and Liu (2023) (28), China	Phenomenological	Semistructured interviews	Colaizzi’s analysis	Twelve ischemic stroke survivors	Exploring the experience of VRBR training for stroke survivors	3 results: perceived benefits of using VR; perceived obstacles of using VR; needs of using VR for stroke patients
Tornbom and Danielsson (2018) (47), Sweden	Phenomenological	Semistructured interviews	Content analysis	Eight sub-acute stroke survivors	Exploring the treadmill walking experience in non-immersive virtual reality	3 results: Enjoyable experience of using VR treadmill; Fatigue experience of using VR treadmill; Suggestions for improving the VR treadmill experience
Gustavsson et al. (2022) (48) Sweden	Descriptive research	Semistructured interviews	Thematic analysis	Six ischemic and one hemorrhagic stroke survivor	Exploring the experience of stroke survivors after VR training on commercial HTC Vive system	3 results: Playing VR training experience; Perceived benefits and effects after VR training; Personalized VR training
Thomson et al. (2020) (49), Britain	Descriptive research	Semistructured interviews	Thematic analysis	Twelve stroke survivors (no stroke type mentioned)	Exploring the rehabilitation experience of stroke survivors using Nintendo Wii	2 results: The experience of rehabilitation for stroke survivors using Wii; Companion has a positive effect on rehabilitation of stroke patients using Wii
Paquin et al. (2016) (25), Canada	Descriptive research	Semistructured interviews	Content analysis	Ten chronic stroke survivors (≥1 year)	Exploring the experience of stroke survivors using VR	4 results: The experience of playing VR; The effect after using VR rehabilitation training; Professional guidance is required; The future of VR in stroke rehabilitation
Moan et al. (2021) (50), Norway	Descriptive research	Semistructured interviews	Thematic analysis	Mixed two types with one stroke survivor, two ischemic stroke survivors, and one hemorrhagic stroke survivor	Exploring the treadmill walking experience in fully immersive virtual reality	2 results: The experience of using VR treadmill; Recommendations for rehabilitation using VR treadmill
Lehmann et al. (2020) (29), Britain	Phenomenological	Semistructured interviews	Thematic analysis	Five stroke survivors (ischemic or hemorrhagic, including two right and three left hemiparesis)	Exploring the experience of the non-immersive virtual reality system You Grabber (YG)	2 results: Medical and clinical elements (general experience of YG, expectations, feedback, arm function of YG, professional physiotherapist help); Social and personal elements (fatigue and positive experience of YG)
Amaya et al. (2018) (51), Britain	Descriptive research	Semistructured interviews	Thematic analysis	Twenty stroke aphasia survivors	Exploring the experience of stroke aphasia survivors using virtual reality EVA Park	3 results: A fun experience after using EVA Park; Perceived benefits of using EVA Park; Perceptual barriers after using EVA Park
Pallesen et al, (2018) (30), Denmark	Phenomenological	Semistructured interviews	Giorgi’s analysis	Six sub-acute stroke survivors	Exploring the experience of stroke survivors with You Grabber (YG) upper limb training	5 results: Motivational factors for using YG training; Engagement with YG training; Perceive the benefits of using YG training; Personalized YG training; Perceive technical barriers after training with YG; Satisfaction with YG training
Krishnan et al. (2023) (26), America	Phenomenological	Semistructured interviews	Thematic analysis	Eleven stroke survivors (>6 months)	Exploring the suggestions of using sports games for rehabilitation of stroke survivors	2 results: Techniques used by stroke survivors; Perceived rehabilitation benefits and barriers using motor games in stroke survivors
Chen et al. (2020) (52), America	Phenomenological	Semistructured interviews	Thematic analysis	Thirteen chronic stroke survivors	Exploring the experience of stroke survivors on home tele-rehabilitation system	5 results: Improved physical and mental health and social skills; Pleasant and convenient experience; Perceived obstacles factors; Peer attention increases motivation; The future and prospect of the system
Wingham et al. (2015) (53), Britain	Phenomenological	Semistructured interviews	Thematic analysis	Eighteen stroke survivors (no stroke type mentioned;<6 months)	Exploring the experience of arm rehabilitation with Wii	5 results: frequency of using Wii; perceived the effectiveness of the Wii; acceptance of using Wii; Caregivers and social support; Setup and management of using Wii
Marika et al. (2018) (54), Canada	Phenomenological	Semistructured interviews	Thematic analysis	Seven sub-acute stroke survivors	Exploring the experience of stroke survivors using VR	3 results: The usefulness of perceived VRBR; Satisfaction with virtual reality intervention; Aspects of virtual reality that need to be improved
Celinder et al. (2012) (27), Denmark	Phenomenological	Semistructured interviews	Content analysis	Nine stroke survivors (no stroke type mentioned)	Exploring the experience of stroke survivors using VR	3 results: Perceive the relationship between VRBR and the diverse needs of daily life; Perceive the benefits of VRBR; Perceive the hindrance factors of using VR
Suzanne et al. (2024) (55), China	Phenomenological	Semistructured interviews	Thematic analysis	Eighteen ischaemic or haemorrhagic stroke survivors	Exploring the experience of stroke survivors using VR	3 results: Shift in attitudes toward VR technology; Perceptions of VR effectiveness; Practical drawbacks and design recommendations
Lo et al. (2024) (56), Britain	Descriptive research	Semistructured interviews	Thematic analysis	Eighteen stroke survivors (≥12 weeks)	Exploring the experience of stroke survivors using VR	5 results: Influences on the use of virtual reality; Perceptions of pretrial preparation, in-trial support and communication; Device usability and comfort; Factors motivating persistence; Perceived effectiveness of the intervention

VRBR is virtual reality-based rehabilitation; Nintendo Wii is non-immersive virtual reality; HTC Vive system is immersive virtual reality; You Grabber (YG) is immersive virtual reality.

### 4.2 Quality assessment of included studies

Among the 16 included studies, 5 had a quality rating of A, and 11 had a rating of B (see Table 2). Among them, item 6 (whether the researcher own situation is described in terms of cultural background and values) of 10 included studies were evaluated as “no.” Item 7 (whether the researcher influence on the research is described, or the research influence on the researcher) of 2 included studies were evaluated as “no,” the remaining entries were evaluated as ‘yes’.

**Table 2:** Quality assessment of included studies.

Literature	1	2	3	4	5	6	7	8	9	10	Quality
Suai Li and Liu (2023) (28)	Yes	Yes	Yes	Yes	Yes	No	Yes	Yes	Yes	Yes	B
Tornbom and Danielsson (2018) (47)	Yes	Yes	Yes	Yes	Yes	Yes	Yes	Yes	Yes	Yes	A
Gustavsson et al. (2022) (48)	Yes	Yes	Yes	Yes	Yes	Yes	No	Yes	Yes	Yes	B
Thomson et al. (2020) (49)	Yes	Yes	Yes	Yes	Yes	No	Yes	Yes	Yes	Yes	B
Paquin et al. (2016) (25)	Yes	Yes	Yes	Yes	Yes	No	Yes	Yes	Yes	Yes	B
Moan et al. (2021) (50)	Yes	Yes	Yes	Yes	Yes	No	Yes	Yes	Yes	Yes	B
Lehmann et al. (2020) (29)	Yes	Yes	Yes	Yes	Yes	No	Yes	Yes	Yes	Yes	B
Amaya et al. (2018) (51)	Yes	Yes	Yes	Yes	Yes	Yes	Yes	Yes	Yes	Yes	A
Pallesen et al, (2018) (30)	Yes	Yes	Yes	Yes	Yes	No	Yes	Yes	Yes	Yes	B
Krishnan et al. (2023) (26)	Yes	Yes	Yes	Yes	Yes	Yes	Yes	Yes	Yes	Yes	A
Chen et al. (2020) (52)	Yes	Yes	Yes	Yes	Yes	No	No	Yes	Yes	Yes	B
Wingham et al. (2015) (53)	Yes	Yes	Yes	Yes	Yes	Yes	Yes	Yes	Yes	Yes	A
Marika et al. (2018) (54)	Yes	Yes	Yes	Yes	Yes	No	Yes	Yes	Yes	Yes	B
Celinder et al. (2012) (27)	Yes	Yes	Yes	Yes	Yes	Yes	Yes	Yes	Yes	Yes	A
Suzanne et al. (2024) (55)	Yes	Yes	Yes	Yes	Yes	No	Yes	Yes	Yes	Yes	B
Lo et al. (2024) (56)	Yes	Yes	Yes	Yes	Yes	No	Yes	Yes	Yes	Yes	B

1. Whether the philosophical basis is consistent with the methodology; 2. Whether the methodology is consistent with the research question or research objectives; 3. Whether the methodology is consistent with the method of data collection; 4. Whether the methodology is consistent with the representativeness of the data and the method of data analysis; 5. Whether the methodology is consistent with the interpretation of the results; 6. Whether the researcher own situation is described in terms of cultural background and values; 7. Whether the researcher influence on the research is described, or the research influence on the researcher; 8. Whether the research object is typical and adequately represents the research subject and its views; 9. Whether the conclusions are drawn in accordance with current ethical norms; 10. Is the researcher influence on the research, or the researcher influence on the research; 8. Is the subject of the research typical and adequately representative of the subject and its views; 9. Is the research consistent with current ethical norms; 10. Are the conclusions drawn from the analysis and interpretation of the data.

### 4.3 Meta-synthesis

**Figure 2:**
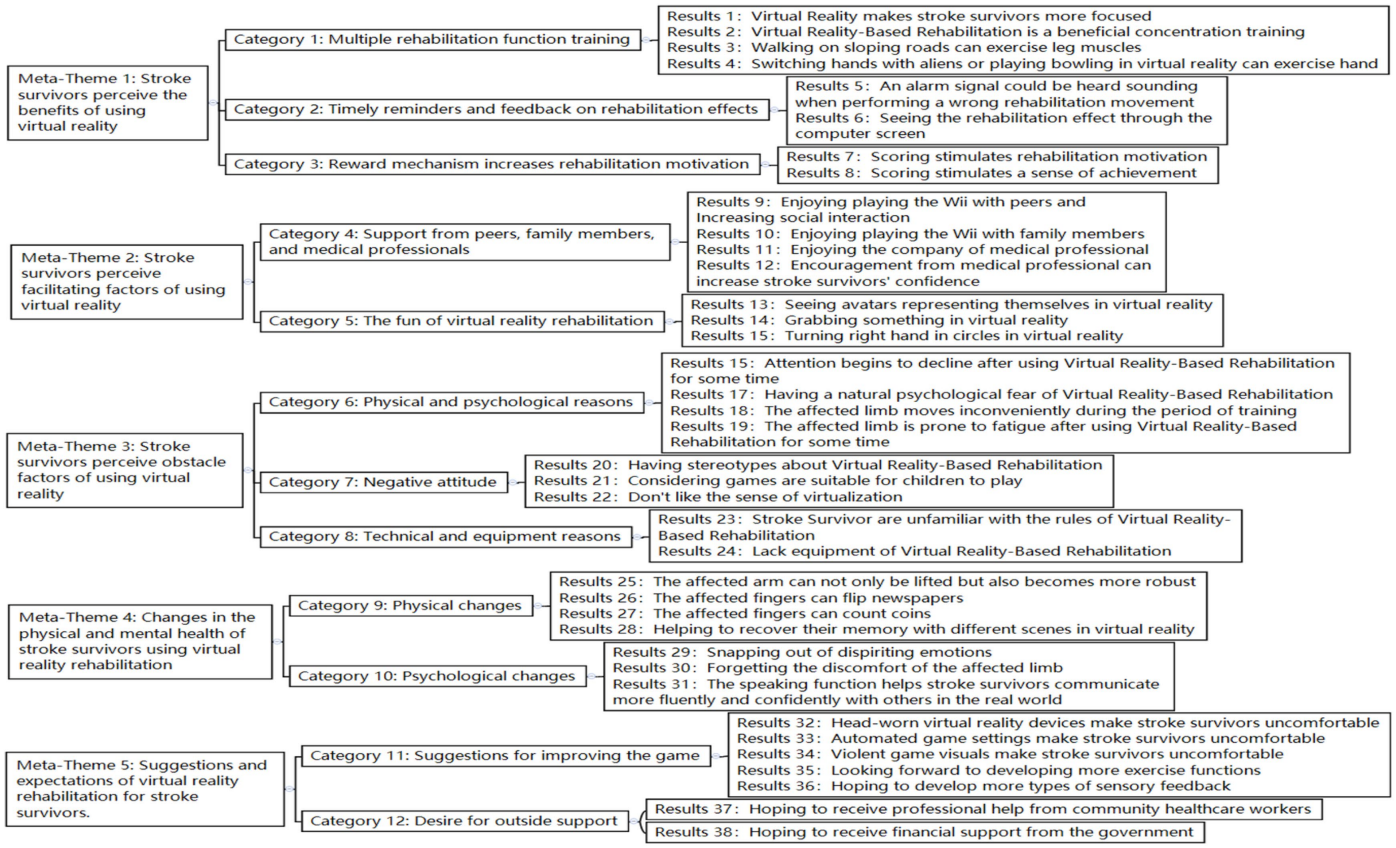
Generalization and integration of research results.

Two researchers interpreted the 16 literature, extracting 38 research findings. Similar results were summarized into 12 new categories, and a comprehensive summary of the five meta-themes was obtained. A detailed generalization and integration of the results are shown in Figure 2.

#### 4.3.1 Meta-theme 1: stroke survivors perceive the benefits of using VR

##### 4.3.1.1 Category 1: diversification of rehabilitation function training

VRBR includes various rehabilitation function training (e.g., concentration training, walking on sloping roads to exercise leg muscles, and stroke survivors switching hands with aliens or using their hands to play bowling in a virtual environment) (10, 18, 21, 22, 25, 26). VR games require focused attention, which is “entirely different than being at the rehabilitation centre” (22). One stroke survivor mentioned, “It’s training in concentration and very beneficial “(25). Others consider walking in VR an effective muscle workout, stating, “Suddenly, there’s a small decline that can tense your muscles differently” (18). In addition to exercising the legs, VRBR involves hand exercises. Aliens in VR could “keep the hand moving” (25) for stroke survivors, and “that game seemed to work” (25). Playing bowling in VR requires “a proper push down” (27) of the hand.

##### 4.3.1.2 Category 2: timely reminders and feedback on rehabilitation effects

In addition to the mentioned rehabilitation training, timely reminders in VR are very useful, and feedback on rehabilitation effects helps stroke survivors correct their rehabilitation movement (10, 23). One participant noted, “When you make a mistake, you can hear the alarm signal, you register it” (23). Another stroke survivor stated, “I think VR rehabilitation is excellent; it can show the rehabilitation effect through a computer screen. I just did the right actions” (10).

##### 4.3.1.3 Category 3: reward mechanism increases rehabilitation motivation

Stroke survivors reported that their motivation and sense of achievement increased as their VR scores increased (19, 26, 28). As one person expressed, “You get motivated to go down there” because “beating your record and getting more and more points” can “motivate you for the next session” (26). Seeing points “went from 3,000 to 30,000” is “some sort of enjoyment in that “(19).

#### 4.3.2 Meta-theme 2: stroke survivors perceive facilitating factors of using VR

##### 4.3.2.1 Category 4: support from peers, family members, and medical professionals

Stroke survivors expressed that playing Wii with family members or peers enhances social interaction, encourages medical professionals to answer questions promptly, and increases their confidence (10, 20, 21). One stroke survivor stated, “Sometimes I play with my niece’s son; we play ten-pin bowling. My niece’s daughter will say, it is my shot; it is my shot. My family has all been in and tried it” (20). Another stroke survivor expressed, “I’ve got friends that will play with the Wii. You could spend all night playing” (20). Playing Wii can “lift the mood and give people something to talk about” (20). Additionally, stroke survivors need the support and encouragement of medical professionals. As stroke survivors mentioned, “during the process of recovery, I want doctors and nurses to be with me at all times, and when I have questions, they can answer them promptly” (10). Medical professionals “kept motivating me to keep going. The encouragement was always encouragement” (21).

##### 4.3.2.2 Category 5: the fun of VR rehabilitation

Stroke survivors reported that seeing avatars representing themselves and performing movements such as grabbing something or turning their right hand in circles were novel and exciting (10, 21, 23). One survivor stated, “When participating in the training project ‘Digital Grid’, I could see a virtual animated character representing myself on the screen, which was very interesting” (10). Another expressed, “For me, to work my hands, my right hand, in circles and stuff, that was entertaining for me” (21). With the help of VR, stroke survivors can “grab something and see it working, although the hand is not open,” which is “interesting” (29).

#### 4.3.3 Meta-theme 3: stroke survivors perceive hindering factors of using VR

##### 4.3.3.1 Category 6: physical and psychological reasons

Stroke survivors reported that decreased attention, natural psychological fear, and affected limbs prevented them from using VR for rehabilitation (10, 19, 23). One stroke survivor expressed, “After a while, you notice your concentration is lessening, then you make more mistakes; this can be seen clearly” (23). The affected limb was prone to fatigue after using VRBR. One stroke survivor stated, “I get tired very quickly and feel tired in the whole body” (19). The affected limbs of stroke survivors can move inconveniently during training. Another stroke survivor said, “I find it inconvenient because, at the moment, I can only walk forward, not backward yet” (10). Moreover, the problems of stroke survivors who cannot walk or stand have been resolved, but they “still have a sense of fear during the VR rehabilitation process” (10).

##### 4.3.3.2 Category 7: negative attitude

Some stroke survivors had stereotypes about rehabilitation games in VR, viewing them as suitable for only children, while others disliked the virtual experience (20, 27, 28). One survivor stated, “At first, I thought, oh God no, I mean it’s, how ashamed am I going to use it? I mean it is for children. That’s what I thought, oh gosh, it’s for, it’s a game, it’s something for kids” (20). Another said, “I do not think you can get that [the effect of walking up a hill] with VR or AR” (27). Stroke survivors are more willing to interact with the natural world; one mentioned, “Doing my dusting and polishing and things like that, I am, probably look around now and see a cobweb is giving me more satisfaction than sitting down playing that [Wii]” (28).

##### 4.3.3.3 Category 8: technical and equipment reasons

Unfamiliarity with rehabilitation rules and insufficient equipment have limited the use of VR rehabilitation among stroke survivors (28, 30). In the beginning, the stroke survivor expressed, “I found it hard to figure out what it was that gave points the rules of the game? Uh! What is it that gives points, what is it I have to go after” (25). This confusion renders their use more challenging. Another important reason was that stroke survivors found that “after being discharged from the hospital, they still want to continue receiving rehabilitation training, unlike traditional rehabilitation that can be done anytime, anywhere, VR rehabilitation requires hardware facilities” (10).

#### 4.3.4 Meta-theme 4: changes in the physical and mental health of stroke survivors using VR rehabilitation

##### 4.3.4.1 Category 9: physical changes

Stroke survivors reported substantial physical improvements (e.g., the ability to lift the affected arm and increased strength, and the affected fingers can flip newspapers or count coins, helping recover their memory of different scenes in VR) (18, 19, 21, 29). As the stroke survivor expressed, “The more I trained, the pain went away, and now I can lie on this side—I can turn” (19). Another stroke survivor stated, My arm started getting stronger. I could reach more, you know, and I practiced it. I started reaching the refrigerator with my right hand and doorknobs (29). Moreover, affected fingers can flip newspapers or count coins. One stroke survivor said, “Well, I can turn a page in the newspaper, you know, with my left hand if I have to. I even handle small coins when I pick them for parking. I pulled them out and counted them. I separate them with my left hand” (21). Additionally, different scenes in VR may help stroke survivors to recover their memories. As one stroke survivor said, “It is easier to bring your memory back for a person who has had a stroke. You know, some people used to go to the sea, while others liked to sit in the forests. This helps them to return to their memories” (18).

#### 4.3.5 Category 10: psychological changes

Stroke survivors reported that they experienced many psychological changes (such as overcoming dispiriting emotions and forgetting the discomfort of the affected limb), and the speaking function helped stroke survivors communicate more fluently and confidently with others in the real world (24, 25, 28). As the stroke survivor expressed, “I was feeling emotional after the…stroke, um, because I was not well enough to do anything round the house… but that [the Wii] just perked me up and made me feel useful” (28). Another stroke survivor mentioned, “I felt, especially at the start, discomfort about that (points to her arm). I do not want to say actual pain but discomfort in that bad arm. So, it has helped me forget discomfort and train” (25). Another said, “I’m more fluent and more confident in outside situations” (24). Additionally, another stroke survivor expressed, “I’m not afraid now of saying what I want to say, so the words come out. Thus, confidence is returned. Cos before I was full of it, but now, after the stroke, it all disappeared” (24).

#### 4.3.6 Meta-theme 5: suggestions and expectations for VR rehabilitation from stroke survivors

##### 4.3.6.1 Category 11: suggestions for improving the game

Head-worn VR devices, automated game settings, and violent game visuals made stroke survivors uncomfortable. They expressed a desire for more exercise options and varied sensory feedback (18, 19, 23, 28). Stroke survivors wanted to wear VR devices in a more comfortable position because one stroke survivor stated, “I would like to have the screen dark and just listen to the sounds, but I get a headache from this headband” (18). Stroke survivors disliked the automated game settings or violent game visuals in VR. A stroke survivor expressed, “Sometimes the Wii would decide that you were going into the sand or going out, and there’s nothing you can do about it” (28). Another stated, “Once, one had a bow, and then you were supposed to shoot those; it’s not allowed really, to shoot someone to death” (18). They proposed improvements in VRBR. As one stroke survivor mentioned, “I would have wanted to put it in more sense! You can have a fan that blows and breezes little over your face as you walk and smell if you go past a lilac hedge, that would have been lovely” (18). Another stroke survivor said, “So in a sense, I was hoping it offers new exercise options, and in my opinion, that is the case” (23).

##### 4.3.6.2 Category 12: desire for outside support

Stroke survivors expressed a desire for guidance on the standard rehabilitation programs from community healthcare workers. As one stroke survivor mentioned, “I was a person who was intent on getting better, and I investigated the internet, did all of these searches, to try and find different ways I could get better. I did not learn about the standard rehabilitation program until approximately 1 year after my stroke. And to me, that’s lost time” (21). Additionally, some stroke survivors discontinued their internet connections because of financial constraints. As one stroke survivor mentioned, “I have a computer, but I gave up my internet for financial reasons” (26). They hope to receive financial support from the government.

### 4.4 ConQual quality evaluation of meta-theme results

Of the 38 extracted research findings, 31 were considered “equivocal.” Among the 14 included studies, 3 assessed the dependability of the three positive responses (Table 3).

**Table 3:** ConQual quality evaluation of meta-theme results.

Meta-theme	Dependability	Credibility	ConQual ratings	Explanation of ratings
Stroke survivors perceive the benefits of using virtual reality	Medium	High	Medium	Nine studies were included, of which two were downgraded by one level in reliability by including three “positive answers.” Eight research findings were “unequivocal,” and credibility was maintained at a high level.
Stroke survivors perceive the facilitating factors of using virtual reality	Medium	High	Medium	Four studies were included, of which one was downgraded by one level in reliability by including three “positive answers.” Seven research findings were “unequivocal,” and credibility was maintained at a high level.
Stroke survivors perceive the hindering factors of using virtual reality	Medium	High	Medium	Seven studies were included, of which three were downgraded by one level in reliability by including three “positive answers.” Nine research findings were “unequivocal,” and credibility was maintained at a high level.
Changes in the physical and mental health of stroke survivors using virtual reality rehabilitation	Medium	Medium	Low	Seven studies were included, of which two were downgraded by one level in reliability by including three “positive answers.” Seven research findings had a mix of “unequivocal” and “equivocal,” so the credibility was downgraded by one level.
Suggestions and expectations of virtual reality rehabilitation for stroke survivors	Medium	High	Medium	Five studies were included, of which one was downgraded by one level in reliability by including three “positive answers.” Seven research findings were “unequivocal,” and credibility was maintained at a high level.

## 5 Discussion

This review provides qualitative evidence of stroke survivors’ experiences with VR rehabilitation, focusing on their needs and perspectives.

### 5.1 The use of VRBR is a complex perceptual change for stroke survivors

This review revealed that some stroke survivors reported improved physical, cognitive, speech, and psychological health through functional exercise with VRBR. This finding aligns with Blazquez-Gonzalez’s study, which indicated that VR could improve depression symptoms in stroke survivors (31), and Demeco’s study, which demonstrated that fully immersive VR improves upper limb flexibility, gait performance, and dynamic balance (32). However, negative attitudes and psychological fear hindered stroke survivors who used VRBR. The underlying causes of this complex perceptual variation may be related to individual differences in stroke survivors (e.g., personality, hobbies, culture, stroke type and location, and other factors). Sports games, such as bowling, are included in the VRBR content; this review found that stroke survivors who enjoyed sports quickly adapted to the intensity of rehabilitation. Stroke survivors may be more receptive to using VRBR in familiar environments, such as at home with family and friends. Some stroke survivors have stereotyped the use of the VRBR, believing that its games are intended for children.

### 5.2 The effect of rehabilitation motivation on the use of VRBR

Motivation is a critical component of a successful rehabilitation program (33). Several factors may influence the use of VRBR among stroke survivors. For example, reward strategies may encourage participation, as many stroke survivors reported that increasing grades motivated them. However, poor upper limb motor function may hinder stroke survivors’ motivation to participate in rehabilitation (34). Additionally, marital intimacy (35) affects the rehabilitation motivation of stroke survivors. Maintaining a strong motivation for rehabilitation improves stroke survivors’ activities of daily living (36), enhancing their quality of life and well-being. Therefore, several potentially relevant factors should be considered when engaging stroke survivors in functional exercises using a VRBR to stimulate motivation and adherence.

### 5.3 External support and assistance are important in the success of using VRBR

This review found that family members, peers, healthcare professionals, and government financial support are important external factors in using VRBR by stroke survivors. Family members and friends offer strong emotional support by accompanying stroke survivors during VRBR sessions. This companionship may increase interpersonal interactions and help restore social connections for stroke survivors. However, the extent and nature of this support may vary depending on cultural norms and healthcare system structures. In regions with limited healthcare resources, informal caregivers often play a central role, whereas in systems with well-established rehabilitation infrastructures, formal healthcare professionals tend to be the primary providers of support (37). Stroke survivors view expert guidance and encouragement from healthcare professionals as key forces in sustaining VRBR use. Studies have shown that music can ameliorate subjective anxiety caused by VR (38), suggesting that integrating calming elements such as music into VR sessions could improve user experience, particularly when fear or discomfort arises.

### 5.4 Optimize VRBR’S content settings and function modes and improve user experience

This review found that violent game visuals, uncomfortable head-worn VR devices, and complex rehabilitation rules are unsuitable for stroke survivors. This finding is similar to Sandra’s study in which a few older adults actively chose violent game visuals (39). Simplicity and ease of understanding are critical factors in older adults’ willingness to use new technology (40). Existing VR rehabilitation content settings and functions may not meet the needs of stroke survivors. Feasibility testing of sports games helps develop more features to meet user needs (41). Feasibility tests can be required the joint engagement of patients with post-stroke aphasia, healthy volunteers, healthcare professionals, and VR experts (42). Therefore, it is recommended that stroke survivors participate in feasibility tests to optimize the content settings and functional modes of VRBR.

## 6 Implications for research and practice

Currently, personalized exergames for in-home rehabilitation after stroke are already in the pilot phase (43). To enhance the usability and acceptance of VRBR systems, future development should incorporate participatory design methods, where stroke survivors are actively involved in co-designing interfaces, interactions, and content. This approach ensures that VRBR platforms align with users’ physical abilities, cognitive capacities, and emotional needs, thereby increasing engagement and adherence. At the same time, technology developers should optimize the VRBR support system (e.g., enriched content and improved functionality) and develop more comfortable virtual training equipment. Reward strategies have been identified as an effective way to increase rehabilitation motivation (44). In the future studies, healthcare professionals and rehabilitation therapists should conduct longitudinal trials of the effectiveness of VRBR interventions tailored based on meta-synthesis findings. Such trials could assess outcomes including motor recovery, psychological well-being, and reintegration into daily living over extended follow-up periods. Lin et al. (45) suggests that stroke survivors have numerous unmet needs when transitioning from hospitalization to the community or home. Thus, healthcare professional should actively promote the integration of VRBR into the community and at home to create a continuum of care. Healthcare professionals and rehabilitation therapists should continue exploring the relationship between reward mechanisms, motivation, and long-term adherence. Finally, stroke survivors should receive high attention from relevant government departments (46). Policymakers should prioritize improving accessibility to VR technology, especially in under-resourced settings, and strategies may include subsidizing VR equipment.

## 7 Limitations

Our meta-synthesis of qualitative studies has certain limitations. Nine studies had a quality rating of B, and one equivocal research finding was identified in 38 research findings. Three included studies had a dependability assessment of three positive responses, which may have created a risk of bias and weakened the overall confidence in our meta-synthesis results. It is important to acknowledge that most of the studies reviewed were conducted in high-income countries, which may limit the generalizability of the findings to other contexts. Beyond these methodological issues, VR-based rehabilitation itself presents broader challenges, such as accessibility barriers (e.g., limited availability of VR systems in clinical or home settings), challenges in aging-friendly design, high costs of VR equipment and treatment (10) and varying levels of technological literacy among stroke survivors, which may hinder widespread adoption and effectiveness.

## 8 Conclusion

VRBR is a complex perceptual change for stroke survivors that requires external support and assistance. Moreover, rehabilitation motivation is an important factor that influences utilization. The VRBR content settings and function modes must be updated to improve user experience.

## Data Availability

The original contributions presented in the study are included in the article/supplementary material, further inquiries can be directed to the corresponding author/s.
